# Mechanical Properties of Rubberised Geopolymer Concrete

**DOI:** 10.3390/ma17051031

**Published:** 2024-02-23

**Authors:** Md Kamrul Hassan, Mohammed Irfan Ibrahim, Sukanta Kumer Shill, Safat Al-Deen

**Affiliations:** School of Engineering and Technology, University of New South Wales, Canberra, ACT 2600, Australia; z5182900@zmail.unsw.edu.au (M.K.H.); s.shill@unsw.edu.au (S.K.S.); s.al-deen@unsw.edu.au (S.A.-D.)

**Keywords:** crumb rubber, geopolymer concrete, rubber treatment, stress–strain curve, modulus of elasticity, rubberised concrete

## Abstract

The environmental impact of non-biodegradable rubber waste can be severe if they are buried in moist landfill soils or remain unused forever. This study deals with a sustainable approach for reusing discarded tires in construction materials. Replacing ordinary Portland cement (OPC) with an environmentally friendly geopolymer binder and integrating crumb rubber into pre-treated or non-treated geopolymer concrete as a partial replacement of natural aggregate is a great alternative to utilise tire waste and reduce CO_2_ emissions. Considering this, two sets of geopolymer concrete (GPC) mixes were manufactured, referred to as core mixes. Fine aggregates of the core geopolymer mixes were partially replaced with pre-treated and non-treated rubber crumbs to produce crumb rubber geopolymer concrete (CRGPC). The mechanical properties, such as compressive strength, stress–strain relationship, and elastic modulus of a rubberised geopolymer concrete of the reference GPC mix and the CRGPC were examined thoroughly to determine the performance of the products. Also, the mechanical properties of the CRGPC were compared with the existing material models. The result shows that the compressive strength and modulus of elasticity of CRGPC decrease with the increase of rubber content; for instance, a 33% reduction of the compressive strength is observed when 25% natural fine aggregate is replaced with crumb rubber. However, the strength and elasticity reduction can be minimised using pre-treated rubber particles. Based on the experimental results, stress–strain models for GPC and CRGPC are developed and proposed. The proposed models can accurately predict the properties of GPC and CRGPC.

## 1. Introduction

Due to a growing demand, the production of automobile tires is continually increasing globally. However, it creates the widespread issue of disposing of worn tires in landfills [1]. The yearly buildup of discarded tires is currently estimated at 1000 million and will potentially be increased to 1200 million by 2030 [2]. The environmental risks caused by heavy metals and contaminants in tires when they are buried in moist landfill soils, leading to the release of poisons into groundwater, further aggravate this problem [3]. In response to the growing environmental risk, rubberised concrete is gaining popularity as a choice for structural applications [4,5]. This involves integrating crumb rubber, which is obtained from discarded tires of trucks and automobiles, into concrete mixes. In recent years, there has been a visible trend in exploring the use of waste rubber, particularly after undergoing crushing treatment, as a substitute for natural aggregates like river sand in concrete preparation [6]. River sand is one of the key ingredients of concrete and a non-renewable natural resource. Therefore, using crumb rubber in concrete offers an eco-friendly solution to reduce the environmental impact associated with tire disposal along with minimising the depletion of natural resources [7].

Some studies have extensively investigated the incorporation of crumb rubber in concrete, focusing primarily on the mechanical behaviours [2,8,9,10,11,12,13]. The quantity, dimensions, and form of rubber aggregate used in concrete have an impact on its mechanical properties. Based on an experimental study, Osama et al. [14] reported that the compressive strength can drop 9% to 20% when 20% of sand is replaced with the rubber particles. Khaloo et al. [15] stated that rubberised concrete with fine rubber particles has acceptable workability, and the replacement of up to 25% mineral aggregates can maintain the acceptable compressive strength of the concrete.

Additionally, the modulus of elasticity (MoE) of rubber-modified concrete is still evolving as a great research interest among researchers. MoE of crumb rubber concrete is observed to be decreased with the increase of rubber content [15,16,17,18,19,20,21,22]. In the past, Zheng et al. [18] stated that a decrease of 5.7% to 28.6% in MoE of ground rubber concrete occurred for a 15% to 45% replacement of coarse aggregates with rubber crumbs. For the crushed rubberised concrete, a decrease in MoE was found at 16.5% to 25.0% compared to the plain concrete. Li et al. [21] reported that a 41.9% decrease in the MoE value was observed with 10% rubber crumb content. Another study by Xie et al. [20] highlighted a 56.3% decrease in MoE when 16% of fine aggregates were replaced with rubber, using recycled concrete aggregates as coarse aggregate. Given the variation in research outcomes, establishing an accurate relationship between MoE and rubber content in the concrete is challenging.

To reduce the strength loss of crumb rubber concrete (CRC), pre-treatment of rubber particles has emerged as an effective approach, and researchers have explored various methods to enhance the compressive strength of rubber concrete [10,23,24,25]. The chemical pre-treatment of rubber, as reported in an experimental study [16], has shown improved adherence and mechanical resistance compared to rubber concrete without pre-treatment. Pham et al. [26] identified that pre-treatment of rubber with NaOH was a key factor contributing to improved adhesion of rubber particles to other ingredients in the concrete mix. Hence, pre-treatment is an essential phase in producing CRGPC, as it significantly affects the bonding of rubber particles with cement paste [26,27,28,29,30,31,32,33]. Khalid Battal Najim [34] experimentally studied the effect of different CR pre-treatment methods, such as water washing, NaOH pre-treatment, cement paste and mortar pre-coating, etc. Raghavan [35] produced high-strength concrete using NaOH solution with favourable results. 

Geopolymer binder is considered an environmentally friendly alternative to OPC, as it generates 70% less greenhouse gas [36,37]. Even though geopolymer concrete is a viable building material, research on rubberised geopolymer concrete is still limited compared to that on rubberised OPC concrete. Like plain concrete, the compressive strength of GPC is an important design parameter. Studies shows that the increase in the concentration of sodium hydroxide (NaOH) in terms of molarity increases the compressive strength of GPC [38,39]. Therefore, employing NaOH in the pre-treatment of crumb rubber to CRGPC not only increases strength, but also holds the potential for minimising chemical waste. Recent research by Giri et al. [39] shows that the compressive strength of CRGPC can be increased by 49% after increasing the NaOH concentration from 10 M to 14 M. Luhar et al. [40] reported that only an 11.66% reduction in compressive strength is observed when 10% of fine aggregate is replaced with crumb rubber treated with NaOH. Moghaddam et al. [41] studied CRGPC by partially replacing fly ash with ordinary Portland cement. The study reported an 8% compressive strength gain for 20% fly ash replacement with OPC and 10% rubber content as a partial replacement of fine aggregate. The modulus of elasticity of CRGPC is rarely studied; Luhar et al. [40] reported that the modulus of elasticity decreased with the increase of rubber content in CRGPC. A similar finding was also reported by Dong et al. [30]. Moreover, the stress–strain behaviour model of GPC focusing on the effect of rubber content is not revealed yet. 

Based on the literature review, the compressive strength of concrete generally reduces with the increase of rubber content. However, pre-treating crumb rubber can significantly reduce the amount of strength loss. To characterise the behaviour of any concrete type, compressive strength, modulus of elasticity, and stress–strain properties are important. Often, the compressive strength of standard concrete is used to calculate most of the other parameters of concrete. However, the current experimental results have not sufficiently validated the method of obtaining the material parameters of crumb rubber concrete, geopolymer concrete, and geopolymer rubber concrete. Hence, an extensive experimental investigation is required. Moreover, there is an insignificant amount of research available on the CRGPC, which is not enough to apply CRGPC in real-life. To date, no material model has yet been able to accurately anticipate the behaviour of CRGPC while being loaded under axial compression.

To achieve the objective of this study, mechanical characteristics, such as compressive strength, stress–strain correlations, and the elastic modulus of rubberised geopolymer concrete were investigated extensively. Results were compared with the existing material models to investigate the models’ suitability in predicting the mechanical properties of CRGPC. Finally, some material models are proposed to predict the properties of CRGPC accurately.

## 2. Materials and Methods

Due to a lack of GPC mix design in existing codes, two GPC mixes were produced using the trial-and-error method. Mix 1 and Mix 2 had an ultimate compressive strength of 44 ± 2 MPa and 26 ± 2 MPa, respectively. These two strength groups of concrete are commonly known as N40 and N25 [42]. For each GPC mix, at least three batches of geopolymer concrete were produced. Each batch contained at least three specimens. After the casting, GPC specimens were kept at room temperature for 24 h before moving into the heat curing phase in an oven at 80 °C for 48 h. After heat curing, specimens were unmoulded and kept at room temperature until the test. In addition to the core mixes (control), geopolymer samples were prepared by replacing 5%, 15%, and 25% of natural fine aggregates with crumb rubber. Specimens were prepared with treated crumb rubber (denoted as TC) and non-treated crumb rubber (denoted as C) to understand the effect of rubber pre-treatment on the mechanical properties. Details of the specimens are shown in Table 1.

Standard compression tests and deformation-controlled compression tests were carried out to investigate compressive strength, stress–strain relationship, and the modulus of elasticity of CRGPC specimens. Two specimens from each batch went through a deformation-controlled compression test, and one of them was subjected to a standard compression test. The failure (cracking pattern) of the cylinder specimens was also observed to have a better understanding of mode of failure of CRGPC. 

### 2.1. Raw Materials and Treatment

#### 2.1.1. Materials

In this study, fly ash and alkaline solutions, such as sodium hydroxide and sodium silicate, riverbed sand as fine aggregate, crushed stone as coarse aggregate, crumb rubber, ground granulate blast furnace slag (GGBFS), super plasticising admixture (ADVA 650), and tape water were used to prepare GPC and CRGPC.

ADVA 650 is a poly carboxylic ether polymer that is widely used in premix and pre-cast industries for maximising concrete strength at an early age. ADVA 650 used in this experiment complies with AS 1478.1 [43] and is compatible with fly ash and blast furnace slag. Admixtures were first mixed with water and then poured into the mixture to ensure uniformity in the mix.

Fly ash used in this study had a fineness of 87% and complied with AS 3582.1 [44]. The chemical composition of the FA is presented in Table 2. 

**Figure 1:**
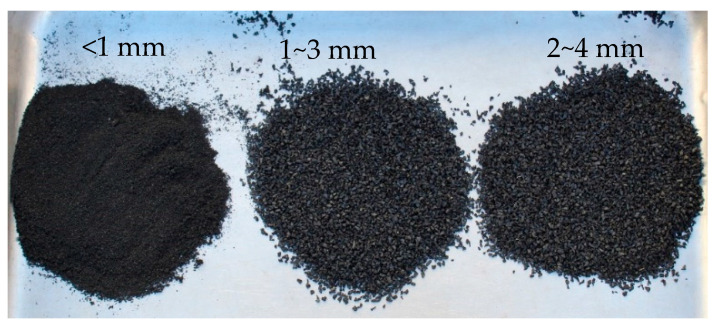
Particle sizes of crumb rubber.

**Figure 2:**
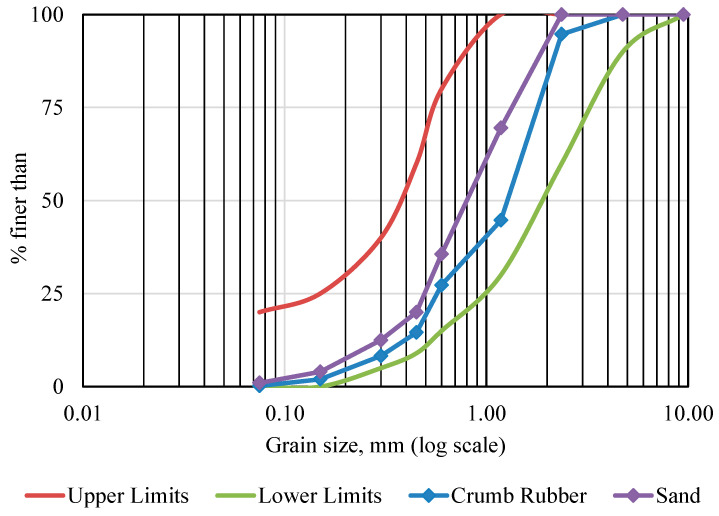
Particle size distribution curve of CR, sand, and allowable limits.

Commercially available mechanically shredded waste tire particles (sizes < 1 mm, 1–3 mm, and 2–4 mm) were used in this study. A volumetric replacement method was applied to replace natural sand with crumb rubber. Three different particle sizes of CR were mixed to obtain a well-graded blending of fine aggregate. The mix ratio of < 1 mm, 1–3 mm, and 2–4 mm CR particles was 8:7:5 (by volume). Figure 1 represents the CR particles used in this experimental research. Sieve analysis was carried out as per AS 1141.0-1999, 1974 [45]. The produced particle size distribution curve is presented in Figure 2. The figure also demonstrates the particle size distribution curve of riverbed sand while showing the upper and lower limits of well-graded fine aggregates. Upper and lower limits for the particle gradation requirements were set by AS 2758.1 [46]. 

#### 2.1.2. Crumb Rubber Pre-Treatment

Mechanically shredded rubber particles usually have a smooth surface, which is not ideal for a good interfacial bond between aggregate and cement paste in concrete. NaOH pre-treatment was adopted in the study as the method of crumb rubber pre-treatment. The molarity effect of sodium hydroxide on gaining compressive strength of geopolymer concrete was experimentally studied by Hardjito and Rangan [47]. In this study, 16M sodium hydroxide solution was used for the pre-treatment of crumb rubber. A 16M NaOH solution was prepared by adding NaOH flakes to water, as shown in Table 3 [48,49].

**Figure 3:**
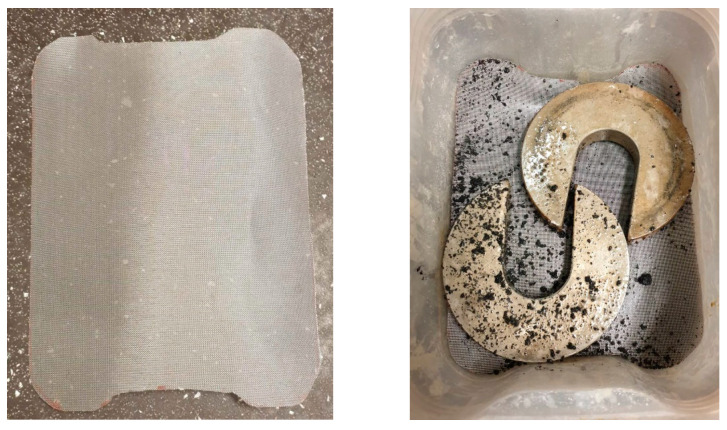
Crumb rubber pre-treatment with sodium hydroxide using a stainless-steel gauze.

CR was soaked into the sodium hydroxide solution for 24 h and air-dried before being used in the concrete mix. Pre-treatment allowed adequate surface roughness, which is essential for better bonding in concrete. As crumb rubber tends to float on a sodium hydroxide solution, a stainless-steel gauze was used all the time to keep the crumb rubbers submerged in the solution, as shown in Figure 3. 

**Figure 4:**
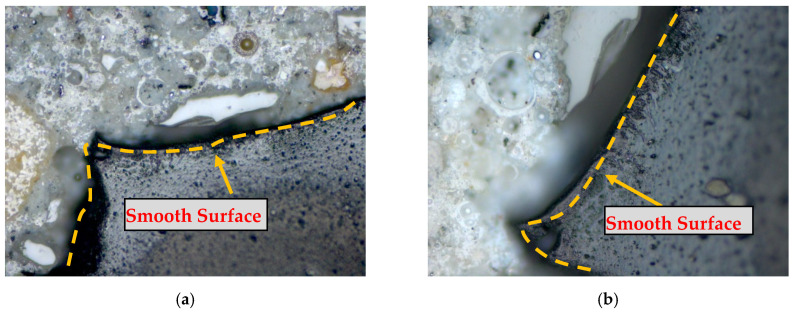
Microscopic analysis of non-treated CR in concrete. (**a**) Example 1 (**b**) Example 2.

**Figure 5:**
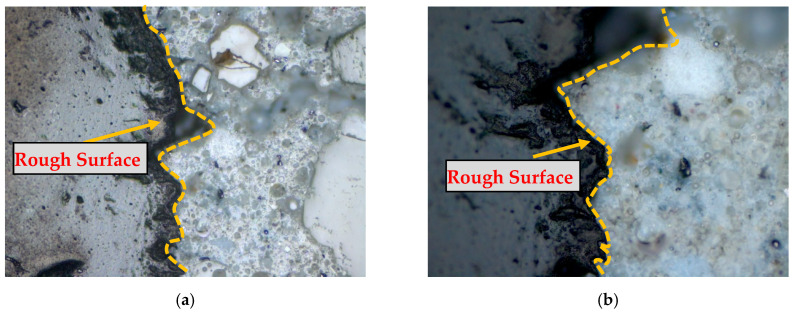
Microscopic analysis of pre-treated CR in concrete. (**a**) Example 1; (**b**) Example 2.

The importance of rubber surface pre-treatment can be more understandable from the microscopic analysis, shown in Figure 4 and Figure 5. Figure 4a,b clearly shows that the surface of non-treated rubber particles is not rough enough to produce a better bond with cement paste in concrete. However, Figure 5a,b shows that the pre-treated CR particles are well bonded with the cement paste of rubberised concrete. A NaOH treatment process cleans the surface of rubber particles and removes other foreign chemicals from the rubber particles. Therefore, to improve the bond between cement paste and crumb rubber, the pre-treatment of rubber particles is imperative.

### 2.2. GPC and CRGPC Mixing

**Figure 6:**
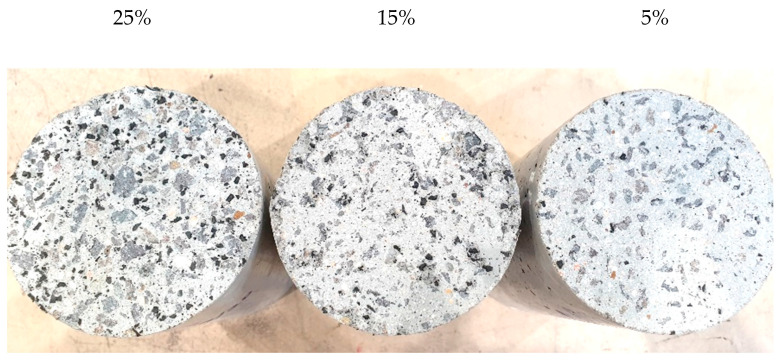
Different rubber content in cylinder specimens.

Based on the trials, the final mix designs used in this study are shown in Table 4 and Table 5. GPC Mix 1 and GPC Mix 2 are considered core mixes in this study. Based on the core mixes, further samples were prepared by replacing 25%, 15%, and 5% of sand with non-treated rubber crumb, while other materials remained the same as the developed core mixes. Pre-treated crumb rubber specimens were prepared, maintaining the same fine aggregate replacement percentages as their non-treated counterparts. When calculating the weight of rubber that is required to replace the fine aggregates in concrete, the specific gravity of both materials was taken into consideration. The specific gravity of crumb rubber and fine aggregate used in this research was 1.15 and 2.6, respectively. Figure 6 shows prepared specimens with different rubber content. Cylinders from left to right contain 25%, 15%, and 5% crumb rubber, respectively.

### 2.3. Curing 

Curing methods significantly affect the compressive strength of GPCs even though they are made from the same mix proportions [50]. In this study, all the samples were cured at 80 °C in an oven for 48 h. After casting, all specimens were kept at ambient/room temperature for 24 h before transferring them to the curing oven. After heat curing, the specimens were transferred to a chamber at room temperature. 

### 2.4. Experimental Setup

**Figure 7:**
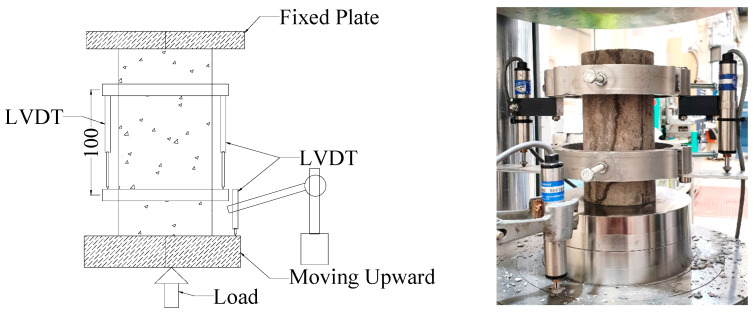
Experimental setup of the stress–strain measurement.

All the tests were conducted at the main civil engineering lab located at the University of New South Wales at the Australian Defence Force Academy. A standard compressive strength test was carried out following the Australian standard AS 1012.9 [51]. Standard cylinders of 200 mm height and 100 mm diameter were used for all tests. Stress–strain behaviour of the test specimens was recorded with a careful setup of two Linear Variable Displacement Transducers (LVDT) on both sides of the specimen. An average strain of these two LVDTs was used as the strain of the specimen, and the gauge length was 100 mm. LVDTs used in these experiments were able to record a movement of 0.001 mm. A third LVDT was used to control the movement of the loading plate. The experimental setup unconfined specimen is shown in Figure 7. 

## 3. Experimental Results and Discussion

### 3.1. Compressive Strength

**Figure 8:**
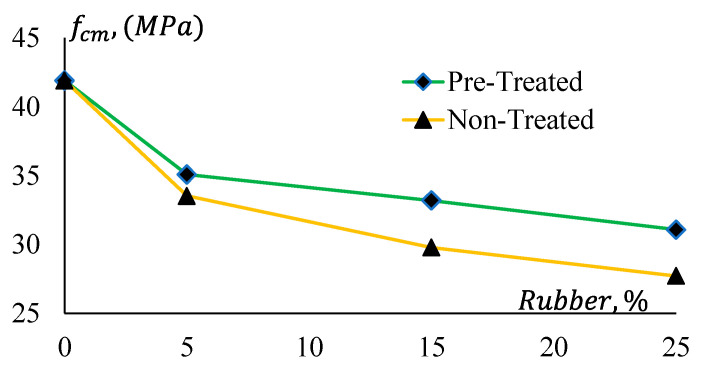
Compressive strengths of Geopolymer concrete Mix 1.

**Figure 9:**
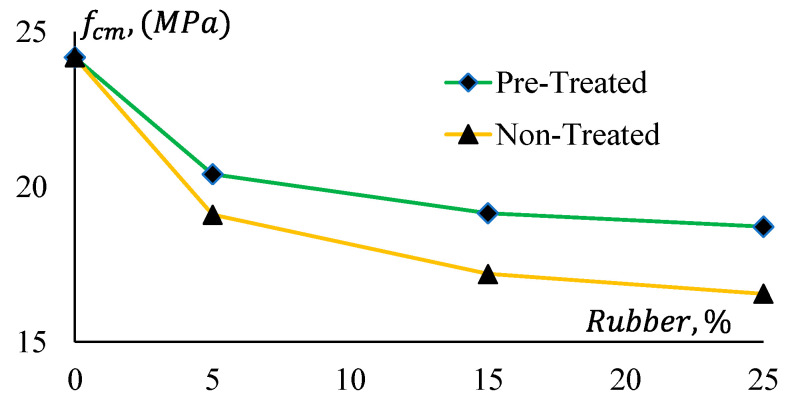
Compressive strengths of Geopolymer concrete Mix 2.

The compressive strengths of all mixes are presented in Table 6. The average of three-cylinder strength results was used to define the compressive strength of a mix. Test results of Mix 1 are graphically presented in Figure 8, and Figure 9 presents the compressive strengths of Mix 2. Here, $f_{cm}$ denotes the mean compressive strength of the samples. The figures show that the compressive strength of both mixes reduces with the increase of rubber content in the mixes. As rubber particles have a lower compressive strength compared to that of natural sand, they reduced the compressive strength of the product. Additionally, the higher the percentage of replacement of natural sand with the rubber particles, the higher the strength loss. It is also observed that the reduction of strength in the pre-treated rubber mixes is lower compared to the counterpart (without treatment). The GPCRC with the treated rubber particles showed better compressive strength compared to that with the untreated rubber particles because of the better bond between the treated rubber and geopolymer paste.

**Figure 10:**
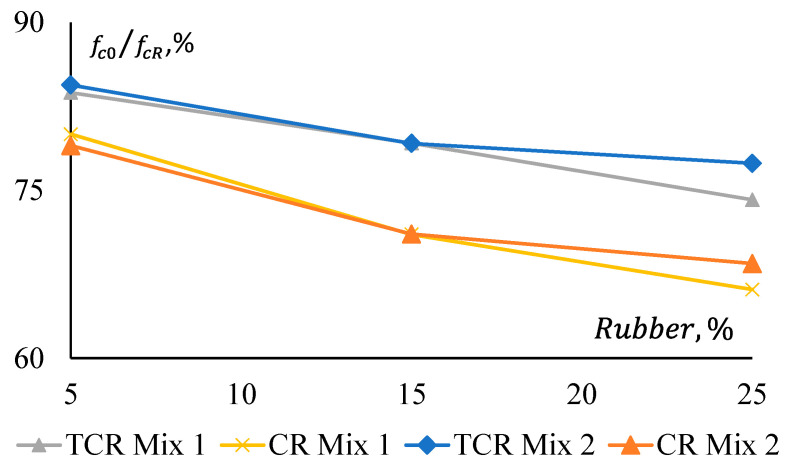
Compressive strength reduction of GPC Mix 1 and CRGPC mixes.

In Figure 10, it is evident that CRGPC with treated rubber provides 4% to 9% higher compressive strength than that of CRGPC with non-treated rubber. An approximately 20% and 16% drop in compressive strength is noticed when 5% non-treated and treated crumb rubber are added to the mixes, respectively.

The reduction in the compressive strength is not observed to be linear. For a 25% replacement of aggregate with non-treated rubber, 34% and 32% strength reductions are observed, whereas a 20% and 21% strength reduction were noticed for only 5% replacement of aggregates in Mix 1 and Mix 2, respectively. 

This indicates that 5% replacement of fine aggregates with rubber has a significant impact on the compressive strength reduction of GPC. However, the rate of reduction of the compressive strength is not the same for higher doses of the crumb rubber. 

**Figure 11:**
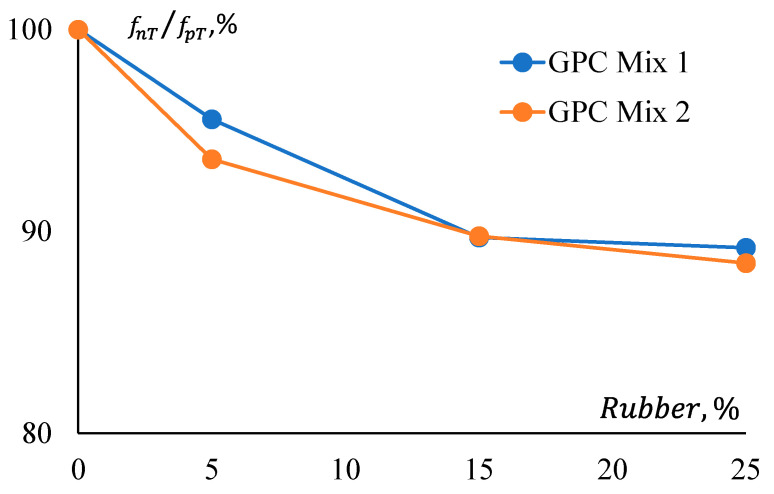
Strength reduction ratio comparison between GPC mixes for rubber treatment.

Figure 11 shows that pre-treatment of rubber has a relatively consistent effect on the strength reduction of both GPC Mix 1 and Mix 2. All CRGPC mixes show a consistent difference in the reduction of their compressive strength for pre-treated and non-treated rubber. Here, $f_{nT}$ denotes the compressive strength of non-treated rubber GPC, and $f_{pT}$ denotes the compressive strength of pre-treated rubber GPC.

### 3.2. Density of Concrete

**Figure 12:**
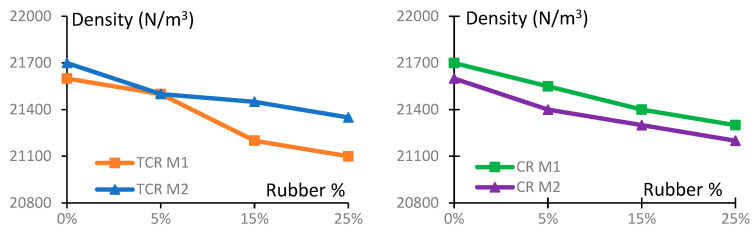
Density of different GPC and CRGPC mixes.

A summary of the density of various GPC and CRGPC mixes used in this research is presented in Table 7 and Figure 12. The test was conducted following AS 1012.5 [52]. It is observed that with the increase of rubber content in the mix, the density of the concrete decreases. As rubber particles possess a lower unit weight compared to that of the natural sand, CRGPC exhibits a decreasing trend in density with the increase of the percentage of rubber particles.

### 3.3. Modulus of Elasticity

**Figure 13:**
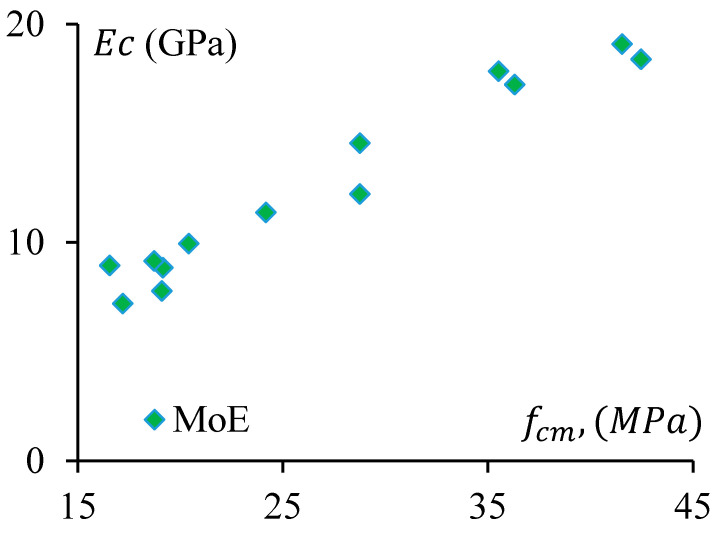
The Modulus of Elasticity of GPC and CRGPC mixes.

The stiffness of a material depends on the elastic modulus, $E_{C}$, of that material. It is a crucial parameter for concrete/geopolymer. The Modulus of Elasticity (MoE) is required to analyse the deflection and seismic performance of concrete structures. In this study, MoE is determined as the secant modulus measured at the 40% stress level of the average compressive strength of a concrete specimen. The MoE of the specimens were obtained from stress–strain curves of the representative samples and presented in Figure 13, which clearly shows that MoE (Ec) of the GPC and CRGPC concrete has a trending relationship with the compressive strength (fcm) of the concrete. The MoE of GPC mixes is determined based on the average MoE of three specimens of the same batch of concrete from the same mix. The MoE values of the different mixes are presented in Table 8.

It is observed that MoE decreases with the increase of rubber content in the concrete mix. When 25% of sand was replaced with pre-treated rubber, it caused a drop in MoE up to 20%, whereas the non-treated crumb rubber resulted in a decrease of 36% of MoE for 25% sand replacement. 

### 3.4. Stress–Strain Behaviour 

**Figure 14:**
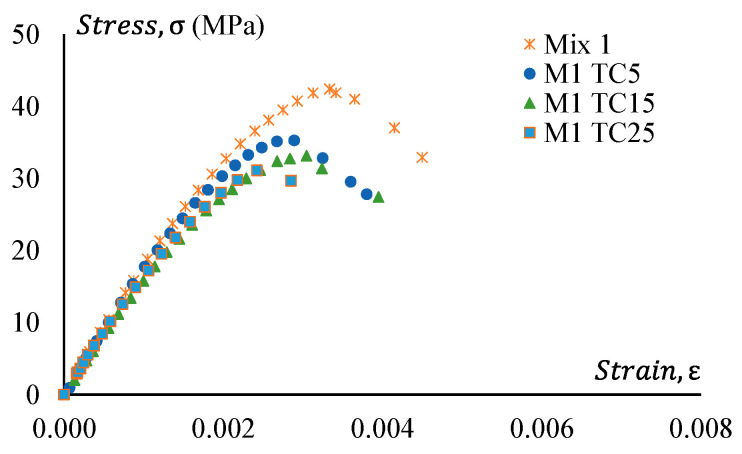
Stress–strain behaviour of GPC Mix 1 and pre-treated CRGPC.

**Figure 15:**
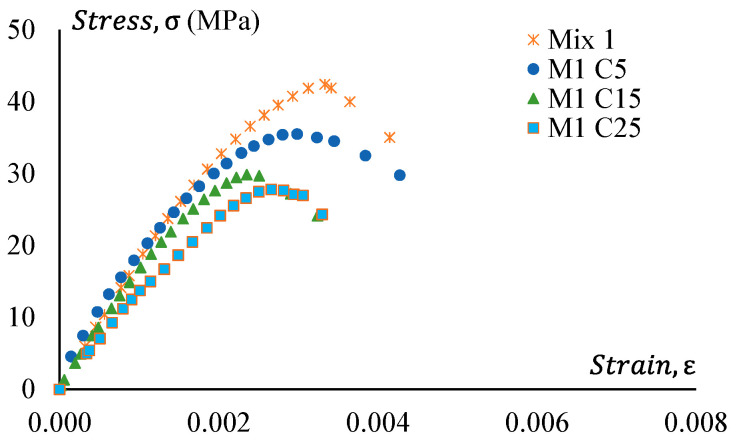
Stress–strain behaviour of GPC Mix 1 and non-treated CRGPC.

**Figure 16:**
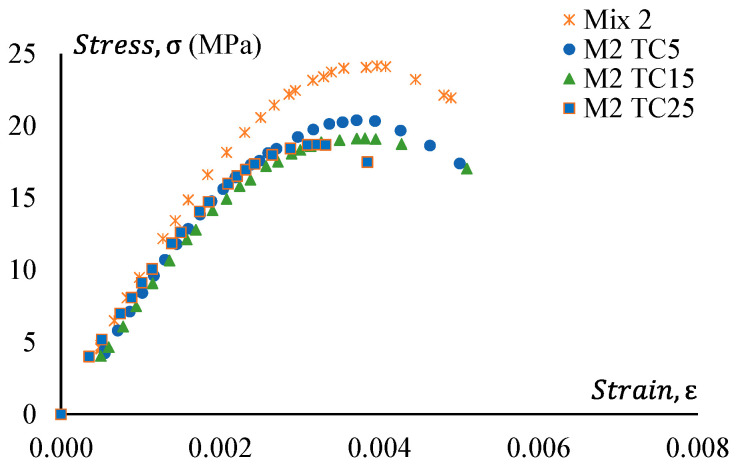
Stress–strain behaviour of GPC Mix 2 and pre-treated CRGPC.

**Figure 17:**
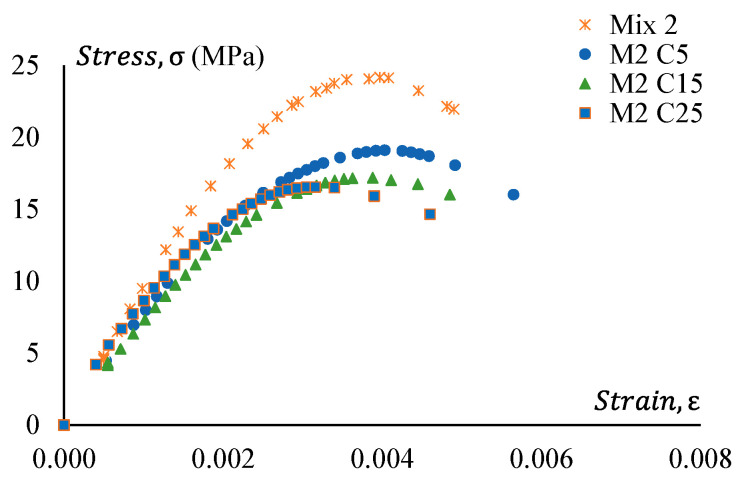
Stress–strain behaviour of GPC Mix 2 and non-treated CRGPC.

Stress–strain curves of all GPC and CRGPC mixes are shown in Figure 14, Figure 15, Figure 16 and Figure 17. Figure 14 shows the stress–strain curve of Mix 1 and various mixes of treated CRGPC originating from the core Mix 1. The result shows that with the increase of rubber content in the mix, the overall strength of the concrete decreases. A decrease in peak strain at the peak load is also noticed, along with the increase in rubber content. 

Figure 15 presents the stress–strain relationship of Mix 1 and various mixes of non-treated CRGPC originating from the core Mix 1. The result presents similar behaviour to the treated CRGPC, except all the values are lower than the pre-treated CRGP. The values of peak strain obtained from the stress–strain curves are presented in Table 9.

Figure 16 shows stress–strain curves of GPC Mix 2 and a treated CRGPC variation of Mix 2. Figure 17 presents the stress–strain relationship of Mix 2 and various mixes of non-treated CRGPC originating from the core Mix 2. The strain value at peak stress of Mix 2 and CRGPC variations of Mix 2 are presented in Table 10.

Based on the results, it is clearly understood that the addition of rubber into the GPC mixes affects the stress–strain behaviour, and pre-treatment of rubber positively contributes to the stress–strain behaviour.

### 3.5. Failure Mode

Under a compressive load, the shear fracture in a concrete cylinder is usually observed to occur diagonally if the interparticle bond in the concrete is good.

**Figure 18:**
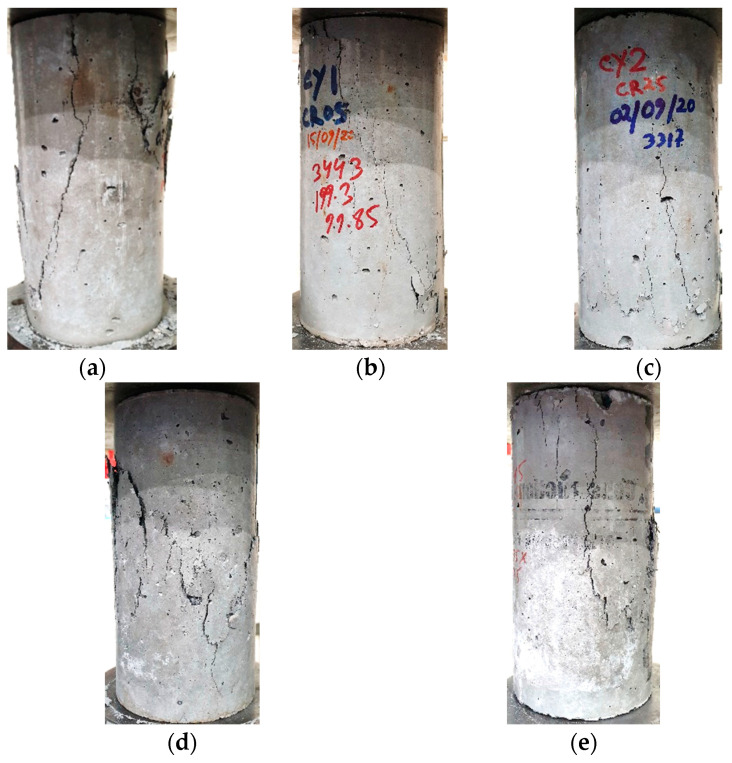
Cylinder failure patterns of GPC and CRGPC. (**a**) GPC Mix; (**b**) CR 05; (**c**) CR 25; (**d**) TCR 05; (**e**) TCR 25.

It is observed from Figure 18a that GPC cylinders primarily failed in shear showing diagonal cracks. However, with the increase of rubber content, the cracks in the cylinder are observed to be steeper for CRGPC. With the increased rubber content, the adhesion between rubber crumbs and binder/paste became weaker. However, Figure 18 shows that cracks in the pre-treated rubber concrete are relatively less steep compared to that on non-treated CRGPC. This indicates that a better inter-particle bond in the pre-treated CRGPC mixes was developed when compared to that of the non-treated counterpart. Photos of the mode of failure of two specimens from each mix are presented in Figure 18. 

## 4. Comparison with Codes and Models

### 4.1. Modulus of Elasticity (MoE) Comparison

The MoE of GPC and CRGPC obtained in the study was compared with the same calculated using the Australian Standard (AS 3600-2018, clause 3.1.2) [53] and the American Concrete Institution (ACI) 363R (1992). Although AS 3600-2018 and ACI-363R 1992 have developed the equations for MoE for OPC concrete, they were used in the study.

Moreover, Mahdi Nematzadeh et al. [54] proposed an equation to predict the MoE of OPC concrete. In the past, some researchers also attempted to develop equations for the determination of MoE of GPC. For instance, Sreenivasulu Chitrala et al. [55] proposed an expression based on regression analysis of experimentally obtained data from GPC concrete.

**Figure 19:**
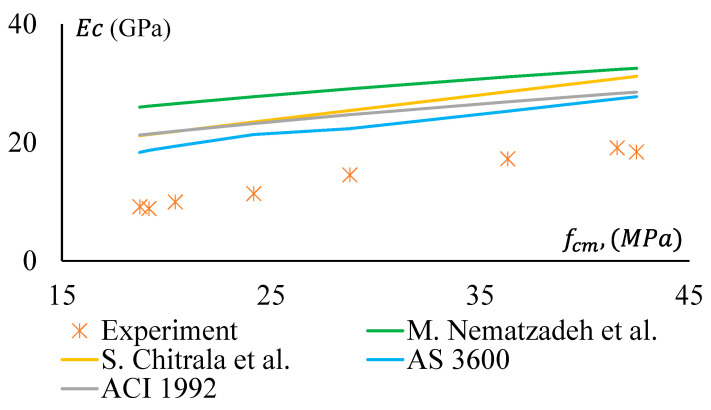
Modulus of Elasticity versus compressive strength of GPC and pre-treated CRGPC [53,54,55,56].

**Figure 20:**
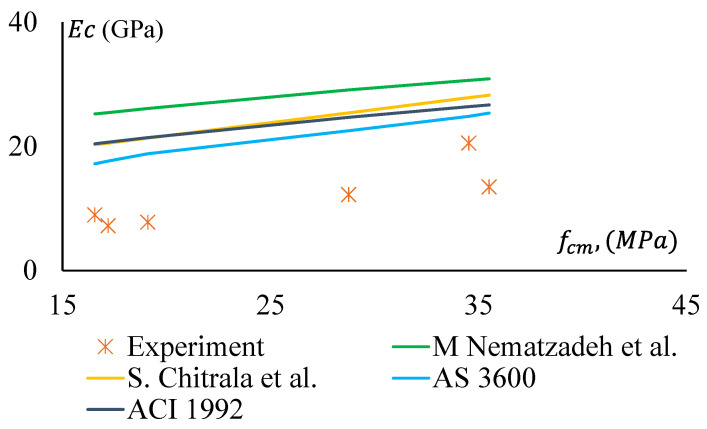
Modulus of Elasticity versus compressive strength of GPC and non-treated CRGPC [53,54,55,56].

Figure 19 and Figure 20 show a comparative study among the MoE of GPC, treated CRGPC, and non-treated CRGPC of this study and the same obtained from AS 3600-2018 and ACI-363R 1992 [53,56]. This shows that the measured $E_{C}$ values of the experimental samples are substantially lower than the predicted value calculated using AS 3600 and ACI models. Hence, AS 3600 (2018) and ACI (1992) models are not suitable for determining the MoE of GPC. The proposed models of Sreenivasulu Chitrala et al. and Mahdi Nematzadeh et al. also provide much higher values than the measured values. Therefore, these models cannot be suitably used to determine the MoE of geopolymer and CRGPC.

For non-treated CRGPC, Figure 20 shows that all the models considered in the study provide significantly higher MoE values compared to the measured values. 

A decrease in the MoE was observed with the increase of rubber content in the mixes; the findings are similar to many recent studies conducted by others [15,17,18,19,20,21,22].

### 4.2. Stress–Strain Behaviour Comparison

A well-established model for the stress–strain relationship of CRGPC is not readily available. Consequently, existing models of stress–strain relationships are used in this study.

#### 4.2.1. Existing Models

Hardjito [57] highlighted that the stress–strain behaviour of GPC is similar to the behaviour of OPC. In his study, he showed a good match of the stress–strain relationship for GPC with the analytical model proposed by Collins et al. [58]. This model was also endorsed by the research of Chitrala et al. [55] in the stress–strain behaviour analysis of GPC. 

Consequently, Popovics [59] proposed a model, which was developed for OPC. The model also has a close match with the results of GPC mixes obtained in this research. In recent years, Noushini et al. [60] proposed a stress–strain relationship model for fly ash-based geopolymer concrete. Additionally, the *fib* model code 2010 [61] describes the stress–strain relationship for short-term uniaxial compression.

#### 4.2.2. Comparison with Existing Models

**Figure 21:**
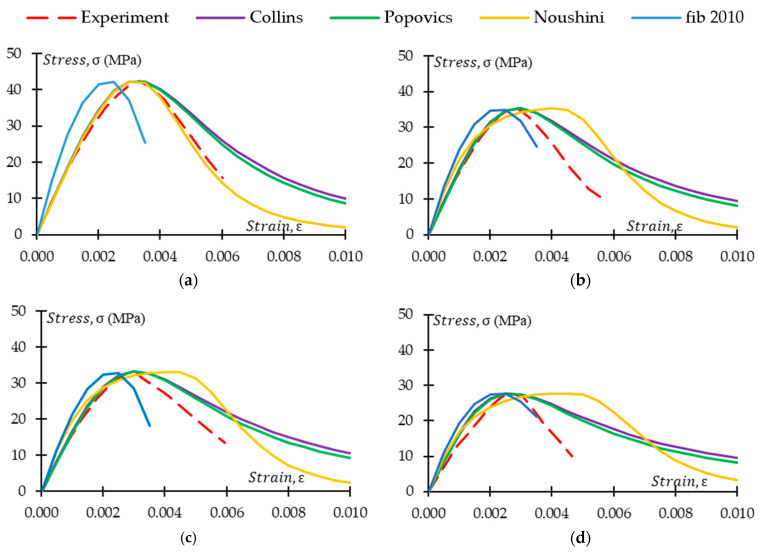

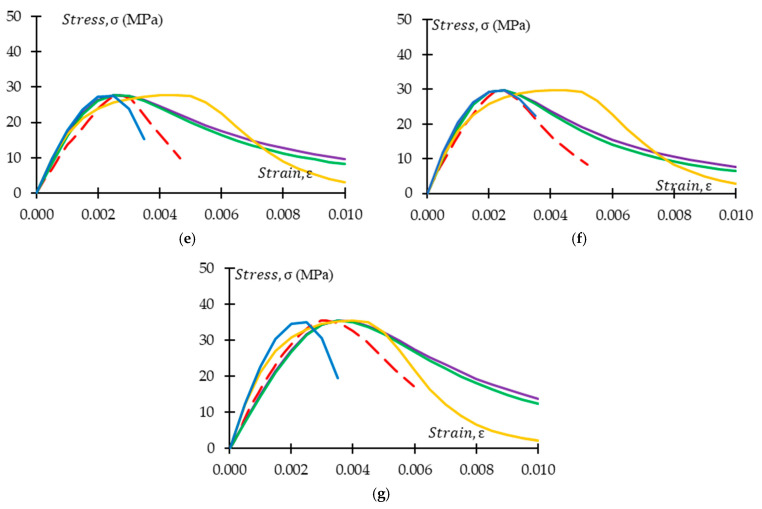
Stress–strain model comparison for GPC Mix 1 with pre-treated CRGPC and non-treated CRGPC mixes. (**a**) M1 stress–strain comparison with the model of Collins, Popovics, Noushini, and fib 2010; (**b**) M1 TC05 stress–strain comparison with the model of Collins, Popovics, Noushini, and fib 2010; (**c**) M1 TC15 stress–strain comparison with the model of Collins, Popovics, Noushini, and fib 2010; (**d**) M1 TC25 stress–strain comparison with the model of Collins, Popovics, Noushini, and fib 2010; (**e**) M1 C25 stress–strain comparison with the model of Collins, Popovics, Noushini, and fib 2010; (**f**) M1 C15 stress–strain comparison with the model of Collins, Popovics, Noushini, and fib 2010; (**g**) M1 C05 stress–strain comparison with the model of Collins, Popovics, Noushini, and fib 2010.

**Figure 22:**
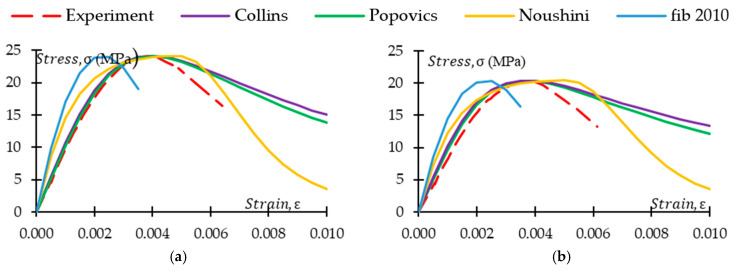

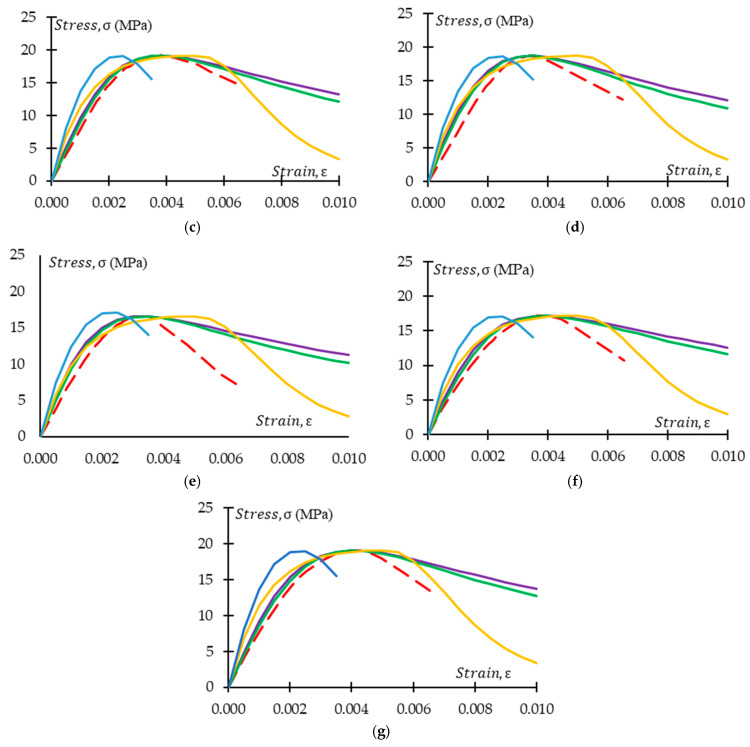
Stress–strain model comparison for GPC Mix 2 with pre-treated CRGPC and non-treated CRGPC mixes. (**a**) M2 stress–strain comparison with the model of Collins, Popovics, Noushini, and fib 2010; (**b**) M2 TC05 stress–strain comparison with the model of Collins, Popovics, Noushini, and fib 2010; (**c**) M2 TC15 stress–strain comparison with the model of Collins, Popovics, Noushini, and fib 2010; (**d**) M2 TC25 stress–strain comparison with the model of Collins, Popovics, Noushini, and fib 2010; (**e**) M2 C25 stress–strain comparison with the model of Collins, Popovics, Noushini, and fib 2010; (**f**) M2 C15 stress–strain comparison with the model of Collins, Popovics, Noushini, and fib 2010; (**g**) M2 C05 stress–strain comparison with the model of Collins, Popovics, Noushini, and fib 2010.

It is crucial to compare the existing models with experimental results of the study to fully understand the models’ effectiveness in explaining the stress–strain behaviour of CRGPC. Figure 21 and Figure 22 illustrate the comparison of experimentally obtained stress–strain data with the various existing models. Figure 21 presents stress–strain curves of GPC Mix 1 and treated variants of CRGPC mixes, while comparing with the models of Collins et al., Popovics et al., Noushini et al., and fib 2010. Figure 22 compares the experimental stress–strain data of non-treated variants of CRGPC mixes originating from GPC Mix 1 with material models.

The models of Collins et al. and Popovics et al. were originally developed for OPC concrete. The above figures show that ascending branches of experimental stress–strain curves match the models of Collins et al. and Popovics et al. However, the slope of the ascending part of the models started to deviate from the experimental results with the increase of rubber content in the mix. The deviation is higher in the non-treated rubber concrete when compared with the pre-treated counterpart. The descending part of the experimentally obtained curves always showed values that were lower than any of the models used. In both models, strain value at peak stress is obtained from the experimental outcomes. Hence, the strain at peak stress has a close match with the experimental results. However, the strain value at peak stress for the models of Noushini et al. and fib 2010 is derived from the equation proposed in the models. From Figure 21 and Figure 22, it is observed that the experimentally obtained stress–strain relationship of the GPC and CRGPC samples did not align with the model of Noushini et al. and fib 2010. The models either overestimate or underestimate the strain at peak stress. From Table 11, it can be observed that for the M1 specimens, the model of Noushini et al. predicted the strain at peak stress up to 91% higher than the experimental results, whereas fib 2010 predicted up to 36% lower than the experimental outcomes. For M2 specimens, the model of Noushini et al. predicted up to 51% higher, and the fib 2010 model predicted up to 49% lower than the experimental results. 

Therefore, the above-mentioned models cannot be suitably used to predict the stress–strain relationship of fly ash-based heat-cured GPC and CRGPC. 

## 5. Proposed Models

### 5.1. Modulus of Elasticity Model

In this study, based on a regression analysis of the experimentally obtained data, a model is proposed to predict the MoE of GPC and CRGPC. The model can be expressed as the following equation.

(1) $$E_{C}=463f_{cm}+188\;(MPa),$$

where $f_{cm}$ is the mean compressive strength of GPC and CRGPC in MPa.

**Figure 23:**
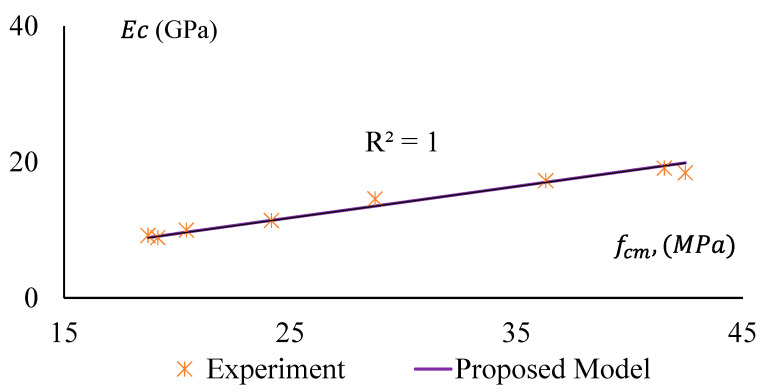
Modulus of Elasticity versus compressive strength of GPC and pre-treated CRGPC for the proposed model and experimental results.

**Figure 24:**
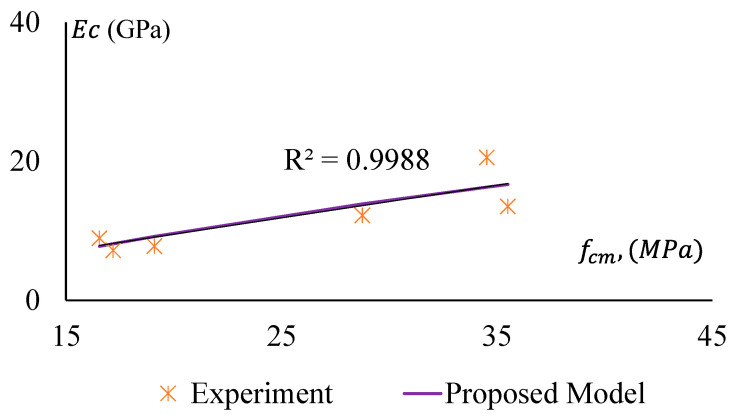
Modulus of Elasticity versus compressive strength of GPC and non-treated CRGPC for the proposed model and experimental results.

Figure 23 and Figure 24 illustrate a well-fitted relationship between the proposed model and experimental data. The data is provided under Table 12. However, for the pre-treated CRGPC, the deviation of experimental results from the predicted model is relatively lower when compared with non-treated CRGPC. Hence, the proposed model can be used to predict the Modulus of Elasticity of heat-treated geopolymer concrete and crumb rubber geopolymer concrete. 

### 5.2. Stress–Strain Model

The suitability of the existing model in predicting the stress–strain relationship of GPC and CRGPC is discussed in Section 4.2. From the comparison of the experimental results with available models, the necessity of a new model to predict the stress–strain behaviour of GPC and CRGPC is understandable. Based on the analysis of the experimental data, a model of the stress–strain relationship for GPC and CRGPC is proposed. The proposed model can be expressed as the equation below.

(2) $$\sigma_{c}=f_{cm}\frac{n(\varepsilon_{c}/{\varepsilon \prime }_{c})}{n-1+{(\varepsilon_{c}/{\varepsilon \prime }_{c})}^{n}}\;(MPa),$$

where n = n_1_ = [1.02 − 1.17 (E_sec_/E_c_)]^−0.95^, if $\varepsilon_{c}$ ≤ ${\varepsilon \prime }_{c}$, for GPC; n_1_ = [1.02 − 1.17 (E_sec_/E_c_)]^−0.85^, if $\varepsilon_{c}$ ≤ ${\varepsilon \prime }_{c}$, for CRGPC; n_2_ = n_1_ + (ϖ + 28 × ζ), if $\varepsilon_{c}$ ˃ ${\varepsilon \prime }_{c}$; ϖ = 17 (12.4 + 0.015 $f_{cm}$)^−0.95^, for GPC; ϖ = 17 (12.4 + 0.015 $f_{cm}$)^−1.5^, for treated CRGPC; ϖ = 17 (12.4 + 0.015 $f_{cm}$)^−1^, for non-treated CRGPC; ζ = 0.83 e^(−911/^$f_{cm}$^)^; E_sec_ = $f_{cm}$/${\varepsilon \prime }_{c}$; ${\varepsilon \prime }_{c}$ = $\frac{2.23\;\times \;10^{-7}{E_{C}}^{1.74}}{{f_{cm}}^{1.98}}$; E_c_ = obtained from Equation (1) $f_{cm}$  and ${\varepsilon \prime }_{c}$ are obtained experimentally.

**Figure 25:**
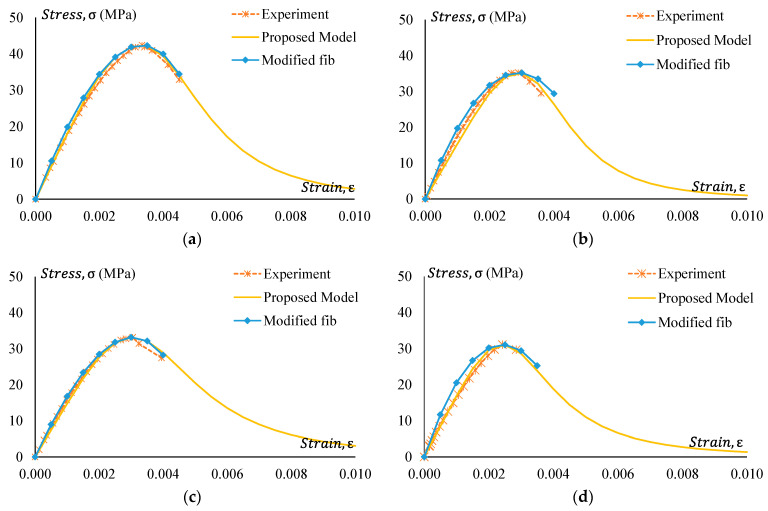
Experimental stress–strain comparison with the proposed model for GPC Mix 1 and pre-treated CRGPC mixes. (**a**) M1 stress–strain comparison with proposed model and modified fib; (**b**) M1 TC05 stress–strain comparison with the proposed model and modified fib; (**c**) M1 TC15 stress–strain comparison with the proposed model and modified fib; (**d**) M1 TC25 stress–strain comparison with the proposed model and modified fib.

**Figure 26:**
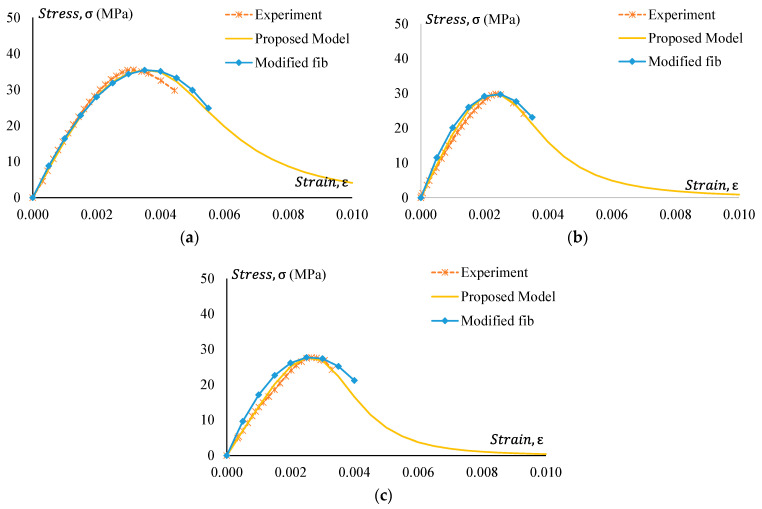
Experimental stress–strain comparison with the proposed model for GPC Mix 1 and non-treated CRGPC mixes. (**a**) M1 stress–strain comparison with proposed model and modified fib; (**b**) M1 C15 stress–strain comparison with the proposed model and modified fib; (**c**) M1 C25 stress–strain comparison with the proposed model and modified fib.

**Figure 27:**
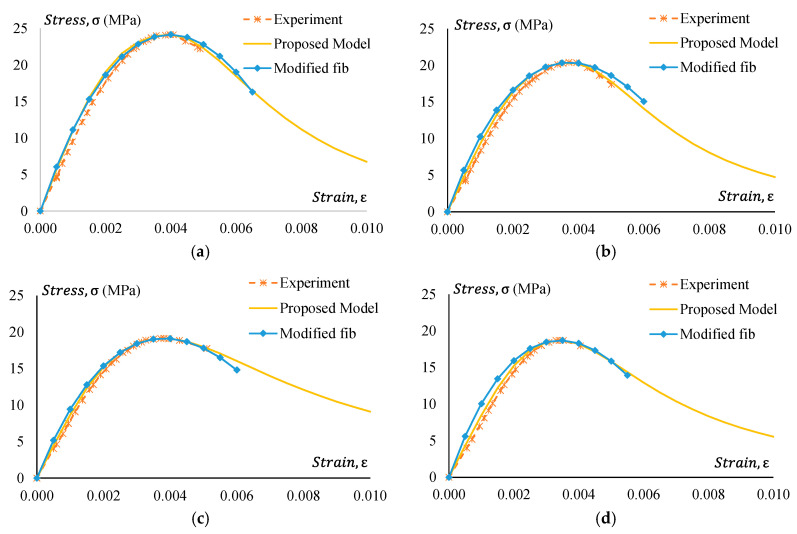
Experimental stress–strain comparison with the proposed model for GPC Mix 2 and pre-treated CRGPC mixes. (**a**) M2 stress–strain comparison with the proposed model and modified fib; (**b**) M2 TC05 stress–strain comparison with the proposed model and modified fib; (**c**) M2 TC15 stress–strain comparison with the proposed model and modified fib; (**d**) M2 TC25 stress–strain comparison with the proposed model and modified fib.

**Figure 28:**
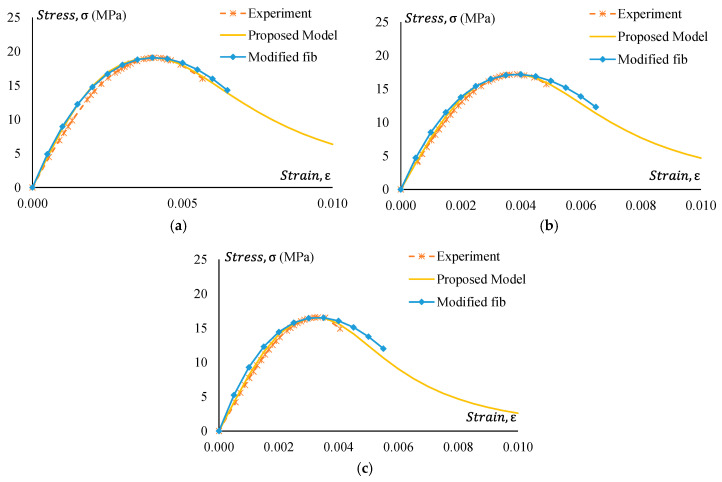
Experimental stress–strain comparison with the proposed model for GPC Mix 2 and non-treated CRGPC mixes. (**a**) M2 C05 stress–strain comparison with the proposed model and modified fib; (**b**) M2 C15 stress–strain comparison with the proposed model and modified fib; (**c**) M2 C25 stress–strain comparison with the proposed model and modified fib.

Figure 25, Figure 26, Figure 27 and Figure 28 present a comparison of the stress–strain relationship between experimental results and the proposed model. It is observed from the mentioned figures that the proposed stress–strain model fits well with the experimental data. To check the suitability of the proposed model, the stress–strain relationship following the fib 2010 model is also plotted in Figure 25, Figure 26, Figure 27 and Figure 28. However, the strain value at peak stress is replaced with the experimental value. Modified fib 2010 fits very well when no rubber is added to the concrete specimens. With the increase of rubber content in the specimens, both the ascending and descending part of the curve deviates from the proposed model and experimental result.

### 5.3. Strain at Peak Stress

Strain at peak stress plays an important role in predicting the stress–strain behaviour of GPC and CRGPC. It is observed from Figure 25, Figure 26, Figure 27 and Figure 28 that when the strain value at peak stress for the fib 2010 model is replaced with the experimentally obtained value, it matches well with the experimental stress–strain behaviour of GPC and CRGPC. Due to a lack of data, a model to predict strain at peak stress was not developed.

## 6. Conclusions

Fine aggregates of geopolymer concrete (GPC) were replaced with pre-treated and non-treated rubber crumbs to produce crumb rubber geopolymer concrete (CRGPC). Emphasis was given to assessing the mechanical properties of CRGPC obtained from the standard compression tests (deformation controlled) on cylinder specimens. Based on the results, new material models are proposed to accurately predict the mechanical properties of CRGPC. The following conclusions have been drawn from this experimental investigation:

1. The compressive strength of CRGPC decreases with the increase of rubber content in the mix. For a 25% fine aggregate replacement with crumb rubber, a 33% strength reduction is observed to happen.
2. Pre-treated rubber particles provided relatively higher compressive strength compared to non-treated rubber particles. Rubber pre-treatment contributed a 4% to 9% increase in the compressive strength.
3. It is also understood that the relationship between the compressive strength and the percentage of replacement of rubber in concrete is not linear for CRGPC.
4. With the increase of rubber content in CRGPC mixes, the Modulus of Elasticity decreases. The decrease is observed to be higher in non-treated CRGPC compared to treated rubber aggregates. For the pre-treated CRGPC, MoE is observed to drop up to 20%; however, for non-treated CRGPC, MoE is observed to drop up to 36%.
5. The CRGPC cylinders showed vertical cracking, with no well-formed cone under the co-axial load.
6. To predict the Modulus of Elasticity and stress–strain of GPC and CRGPC, the existing models for conventional concrete are found to be not suitable. However, the proposed models can reasonably predict the Modulus of Elasticity and stress–strain properties of heat-cured GPC and CRGPC.

## Figures and Tables

**Table 1:** Specimen details of GPC and CRGPC mixes.

Mix ID	Number of Specimens	Mix ID	Number of Specimens
Mix 1	9	Mix 2	9
M1 TC25	9	M2 TC25	9
M1 TC15	9	M2 TC15	9
M1 TC05	9	M2 TC05	9
M1 C25	9	M2 C25	9
M1 C15	9	M2 C15	9
M1 C05	9	M2 C05	9

TC = Treated Crumb Rubber; C = Crumb Rubber.

**Table 2:** Chemical composition of fly ash.

Element	Content (%)
Al_2_O_3_	24.00
CaO	1.59
Fe_2_O_3_	2.87
K_2_O	1.44
MgO	0.42
MnO	0.06
Na_2_O	0.49
P_2_O_5_	0.19
SiO_2_	65.9
TiO_2_	0.915
LOI	1.53

**Table 3:** Mix design of molarity for NaOH [49].

NaOH Solution Molarity	Weight of NaOH Flakes (g/kg)
8	262
10	314
12	361
16	444

**Table 4:** Mix design of GPC Mix 1.

Mix Design of Mix 1 (kg/m^3^)					
Materials	Detail	M1	M1-R25	M1-R15	M1-R05
Fly Ash		365.00	365.00	365.00	365.00
Sodium Hydroxide		49.00	49.00	49.00	49.00
Sodium Silicate		122.50	122.50	122.50	122.50
Water		50.38	50.38	50.38	50.38
Fine aggregate	Sand	535.00	401.25	454.75	508.25
	Rubber	0.00	59.16	35.50	11.83
Coarse aggregate	7 mm	245.00	245.00	245.00	245.00
	10 mm	430.00	430.00	430.00	430.00
	14 mm	555.00	555.00	555.00	555.00
GGBFS		40.00	40.00	40.00	40.00
Super Plasticiser		3.80	3.80	3.80	3.80

**Table 5:** Mix design of GPC Mix 2.

Mix Design of Mix 2 (kg/m^3^)					
Materials	Detail	M2	M2-R25	M2-R15	M2-R05
Fly Ash		445.76	445.76	445.76	445.76
Sodium Hydroxide		63.68	63.68	63.68	63.68
Sodium Silicate		159.20	159.20	159.20	159.20
Water		48.28	48.28	48.28	48.28
Fine aggregate	Sand	571.18	428.39	485.50	542.62
	Rubber	0.00	63.16	37.90	12.63
Coarse Aggregate	10 mm	1243.10	1243.10	1243.10	1243.10
Super Plasticiser		6.73	6.73	6.73	6.73

**Table 6:** Compressive strengths of GPC and CRGPC mixes.

Mix ID	Strength (MPa)	Batch Strength (MPa)	Mix ID	Strength (MPa)	Batch Strength (MPa)
Mix 1	41.53	41.91	Mix 2	25.06	24.18
	41.51			23.47	
	42.69			24.01	
M1 TC05	36.05	35.09	M2 TC05	20.95	20.41
	34.07			20.97	
	35.15			19.31	
M1 TC15	32.92	33.2	M2 TC15	19.73	19.15
	31.51			19.16	
	32.17			18.56	
M1 TC25	31.49	31.08	M2 TC25	18.66	18.72
	30.9			17.58	
	30.85			19.92	
M1 C05	33.42	33.53	M2 C05	20.01	19.1
	33.66			18.28	
	33.51			19.02	
M1 C15	28.98	29.78	M2 C15	16.53	17.19
	29.77			17.91	
	30.58			17.12	
M1 C25	26.67	27.72	M2 C25	16.57	16.55
	28.93			16.51	
	27.56			16.58	

TC = Treated Crumb Rubber; C = Crumb Rubber.

**Table 7:** Density of concrete GPC and CRGPC.

Mix ID	Density (N/m^3^)	Mix ID	Density (N/m^3^)
M1	21,600	M2	21,700
M1 TC05	21,500	M2 TC05	21,500
M1 TC15	21,200	M2 TC15	21,450
M1 TC25	21,100	M2 TC25	21,350
M1 C05	21,400	M2 C05	21,550
M1 C15	21,300	M2 C15	21,400
M1 C25	21,200	M2 C25	21,300

TC = Treated Crumb Rubber; C = Crumb Rubber.

**Table 8:** The Modulus of Elasticity of GPC and CRGPC mixes.

Mix ID	MoE (GPa)	Batch MoE (GPa)	Mix ID	MoE (GPa)	Batch MoE (GPa)
Mix 1	18.44	18.40	Mix 2	11.42	11.37
	18.75			11.67	
	18.01			11.03	
M1 TC05	18.54	19.09	M2 TC05	9.9	9.95
	19.69			9.42	
	19.03			10.52	
M1 TC15	17.28	17.24	M2 TC15	8.96	8.85
	17.79			9.19	
	16.65			8.41	
M1 TC25	14.69	14.55	M2 TC25	9.24	9.16
	14.37			8.85	
	14.59			9.38	
M1 C05	17.33	17.84	M2 C05	7.53	7.78
	17.94			7.52	
	18.24			8.28	
M1 C15	21.27	20.55	M2 C15	7.13	7.21
	20.16			7.48	
	20.22			7.01	
M1 C25	11.94	12.22	M2 C25	9.32	8.95
	12.48			8.47	
	11.88			9.07	

TC = Treated Crumb Rubber; C = Crumb Rubber.

**Table 9:** Strain at peak stress of GPC Mix 1 and CRGPC mixes.

Mix ID	Strain at Peak Stress, (ε)
Mix 1	0.00333
M1 TC05	0.00289
M1 TC15	0.00305
M1 TC25	0.00242
M1 C05	0.00363
M1 C15	0.00235
M1 C25	0.00266

TC = Treated Crumb Rubber; C = Crumb Rubber.

**Table 10:** Strain at peak stress of GPC Mix 2 and CRGPC mixes.

Mix ID	Strain at Peak Stress, (ε)
Mix 2	0.00397
M2 TC05	0.00371
M2 TC15	0.00382
M2 TC25	0.00342
M2 C05	0.00403
M2 C15	0.00388
M2 C25	0.00330

TC = Treated Crumb Rubber; C = Crumb Rubber.

**Table 11:** Experimental results of strain at peak stress comparison with the model of Noushini et al. and fib 2010.

Mix ID	Noushini et al.	Fib 2010
	% Higher	% Lower
M1	4.99	25.00
M1 TC05	38.38	13.51
M1 TC15	31.34	17.91
M1 TC25	85.84	3.25
M1 C05	26.74	36.63
M1 C15	91.41	14.93
M1 C25	69.11	24.84
M2	25.90	49.64
M2 TC05	34.66	46.14
M2 TC15	30.96	47.62
M2 TC25	46.26	41.49
M2 C05	23.41	50.64
M2 C15	28.88	48.45
M2 C25	51.15	39.54

**Table 12:** Elastic modulus of GPC and CRGPC as of the model.

Mix ID	Experiment	Proposed Model	Mix ID	Experiment	Proposed Model
	(GPa)	(GPa)		(GPa)	(GPa)
Mix 1	18.40	19.84			
M1 TC05	19.09	19.41	M1 C05	13.50	16.63
M1 TC15	17.24	16.99	M1 C15	20.55	16.18
M1 TC25	14.55	13.50	M1 C25	12.23	13.50
Mix 2	11.37	11.38			
M2 TC05	9.95	9.64	M2 C05	7.78	9.03
M2 TC15	8.85	9.05	M2 C15	7.21	8.15
M2 TC25	9.16	8.85	M2 C25	8.96	7.85

TC = Treated Crumb Rubber; C = Crumb Rubber.

## Data Availability

Data are contained within the article.
